# Cardiac and intestinal tissue conduct developmental and reparative processes in response to lymphangiocrine signaling

**DOI:** 10.3389/fcell.2023.1329770

**Published:** 2023-12-21

**Authors:** Shreya Kurup, Can Tan, Tsutomu Kume

**Affiliations:** ^1^ Department of Medicine, Feinberg Cardiovascular and Renal Research Institute, Feinberg School of Medicine, Northwestern University, Chicago, IL, United States; ^2^ Honors College, University of Illinois at Chicago, Chicago, IL, United States

**Keywords:** lymphatic vessel, lymphangiogenesis, lymphangiocrine factors, FOXC, reelin, R-spondins

## Abstract

Lymphatic vessels conduct a diverse range of activities to sustain the integrity of surrounding tissue. Besides facilitating the movement of lymph and its associated factors, lymphatic vessels are capable of producing tissue-specific responses to changes within their microenvironment. Lymphatic endothelial cells (LECs) secrete paracrine signals that bind to neighboring cell-receptors, commencing an intracellular signaling cascade that preludes modifications to the organ tissue’s structure and function. While the lymphangiocrine factors and the molecular and cellular mechanisms themselves are specific to the organ tissue, the crosstalk action between LECs and adjacent cells has been highlighted as a commonality in augmenting tissue regeneration within animal models of cardiac and intestinal disease. Lymphangiocrine secretions have been owed for subsequent improvements in organ function by optimizing the clearance of excess tissue fluid and immune cells and stimulating favorable tissue growth, whereas perturbations in lymphatic performance bring about the opposite. Newly published landmark studies have filled gaps in our understanding of cardiac and intestinal maintenance by revealing key players for lymphangiocrine processes. Here, we will expand upon those findings and review the nature of lymphangiocrine factors in the heart and intestine, emphasizing its involvement within an interconnected network that supports daily homeostasis and self-renewal following injury.

## Introduction

The vascular network is comprised of two independent yet cooperative conduits for the transport of fluid connective tissue: the blood and lymphatic systems. The blood circulatory system transports vital metabolites, nutrients, gasses, fluids, hormones, and immune cells to tissues (Potente and Makinen, 2017). Unlike blood circulation, the lymphatic system is an open circulatory system with capillary vessels that can directly absorb fluid from the nearby tissue (Potente and Makinen, 2017). Lymph flows through vessels lined with lymphatic endothelial cells (LECs) that can secrete growth factors, cytokines, and chemokines (Petrova and Koh, 2018). Substances that fall under this paracrine signaling system are collectively referred to as lymphangiocrine factors (Petrova and Koh, 2018). The release of lymphangiocrine factors is contingent on the specific context and tissue conditions, allowing for adaptable lymphatic vessel growth and tissue regeneration in response to injury (Petrova and Koh, 2018). This is particularly important for conditions affecting the heart and intestine that exhibit persistent inflammation, which is a major culprit for cardiomyocyte death and epithelial damage (Luissint et al., 2016; Zhang et al., 2022), whereas bone marrow (BM)-derived progenitors can also directly contribute to the regeneration of both cardiomyocytes (Fukata et al., 2013; Gentek and Hoeffel, 2017) and intestinal epithelium (Rizvi et al., 2006; Davies et al., 2009). The infiltration of neutrophils, macrophages, and other immune cells can induce pathological alterations to lymphatic vessels in the region, preventing the efficient removal of inflammatory factors and triggering a chronic inflammatory state (Henri et al., 2016; Bernier-Latmani and Petrova, 2017). Recent advances in imaging technology paired with studies in mouse models showcase key lymphangiocrine factors that can augment organ tissue growth under pathological states (Liu et al., 2020; Tan et al., 2023a). Understanding the mechanisms by which the modulation of lymphangiocrine factors can assist tissue regeneration could be advantageous in restoring organ function under cardiovascular and intestinal disease. In this review, we explore the latest research on lymphangiocrine factors that can mediate both the maintenance and regeneration of cardiomyocytes or intestinal epithelial cells, while also facilitating therapeutic lymphangiogenesis.

## Function and development of the lymphatic system

The lymphatic system allows for the maintenance of fluid homeostasis throughout the body. Interstitial fluid travels through the button-like adhesion junctions that connect LECs of the lymphatic capillaries (Norrmen et al., 2011; Kalucka et al., 2017; Secker and Harvey, 2021; Angeli and Lim, 2023). The contraction of surrounding smooth muscle cells (SMCs), coupled with luminal valves that prevent backflow, guides the movement of lymph through pre-collector vessels and into larger collecting vessels (Norrmen et al., 2011; Kalucka et al., 2017; Petrova and Koh, 2018; Klaourakis et al., 2021; Secker and Harvey, 2021; Angeli and Lim, 2023). Lymph is continuously filtered at intermittently positioned lymph nodes until it finally reaches the thoracic ducts and replenishes the blood plasma volume (Geng et al., 2021; Secker and Harvey, 2021). Lymphatic vessels are derived from the blood vasculature (Xavier and Podolsky, 2007; Yang et al., 2012; Liu and Oliver, 2019; Lioux et al., 2020). Based on embryonic mouse models, accumulating evidence indicates that the transcription factors COUP-TFII and SOX18 bind Prospero homeobox protein 1 (*Prox1*), and its transcription is necessary for the differentiation of blood endothelial cells (BECs) into LECs (Karkkainen et al., 2004; Francois et al., 2008; Johnson et al., 2008; Yang et al., 2012; Liu and Oliver, 2019; Lioux et al., 2020). Soon after LECs are formed, Vascular Endothelial Growth Factor C/Vascular Endothelial Growth Factor Receptor 3 (VEGF-C/VEGFR-3) signaling allows them to relocate and form bilateral anteroposterior chains of primary lymph sacs and, eventually, the lymphatic plexus (Juszynski et al., 2008; Yang et al., 2012; Zheng et al., 2014; Lioux et al., 2020; Klaourakis et al., 2021). Lymphangiogenesis facilitates the emergence of new lymphatic vessels from the plexus, which remodel into the various components of the lymphatic vasculature (Norrmen et al., 2011; Zheng et al., 2014; Geng et al., 2021; Ujiie and Kume, 2022). The flow of lymph fluid further upregulates *Prox1* expression which, alongside the expression of GATA-binding protein 2 (*Gata2*) and forkhead box C2 (*Foxc2*), induces lymphatic valve formation (Kume, 2015; Ujiie and Kume, 2022). As lymphatics mature and extend across the organ systems, their lymphatic networks become tailored for their distinctive microenvironments (Petrova and Koh, 2018).

## Role of lymphatics in the cardiac microenvironment

Powered by cardiac contractions, lymphatic vessels propel lymphatic fluid from capillaries in the endocardium to collecting vessels in the epicardium, ultimately draining into the venous system via mediastinal lymph nodes (Huang et al., 2017). Over the years, numerous works have covered the structural, functional, and developmental characteristics of the lymphatic vasculature in the heart (Klotz et al., 2015; Huang et al., 2017; Brakenhielm and Alitalo, 2019; Gancz et al., 2020; Klaourakis et al., 2021; Tan et al., 2023b). In mice, lymphangiogenesis is first detected at around E12.5 near the ventral outflow tract (Klotz et al., 2015). *Vegfr3* and *Prox1* expression guides the subsequent formation of the lymphatic plexus, spanning the epicardial layer of the heart (Juszynski et al., 2008; Gancz et al., 2020; Klaourakis et al., 2021). Once fully developed, lymphatic vessels co-express *Prox1*, *Vegfr3*, lymphatic vessel endothelial hyaluronan receptor 1 (*Lyve1*), and podoplanin (*Pdpn*) (Klotz et al., 2015; Tatin et al., 2017). Lymphatic markers are not just valuable tools for labeling lymphatic vessels (Kong et al., 2017). Conditional knockout models involving lymphatic markers have provided valuable insight into the importance of lymphatic vessels in heart development (Karkkainen et al., 2001; Lossi et al., 2019). The deletion of *Prox1* from embryonic LECs shrunk the heart by approximately 33%, and a decrease in *Vegfr3* expression in embryonic LECs also demonstrated a reduction in heart size (Liu et al., 2020). In both cases, the change in cardiomyocyte numbers was not reflected in other cell types within the heart, hinting that lymphatic vessels communicate with cardiomyocytes in a way that is essential for heart development (Liu et al., 2020).

**FIGURE 1:**
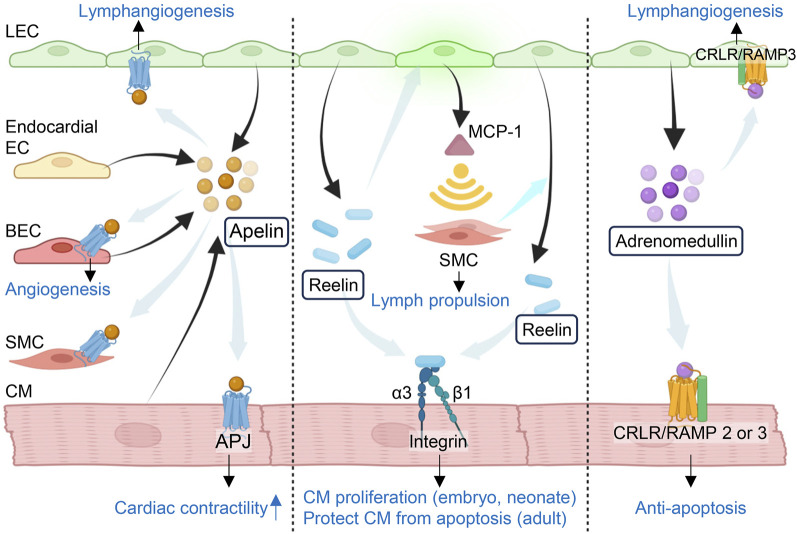
Lymphangiocrine factors in the heart. Cardiac lymphatic endothelial cells express tissue specific signals to maintain or restore cardiac function after injury. Apelin is expressed by lymphatic endothelial cells (LECs) in newly-formed lymphatic vessels, endocardial ECs, blood endothelial cells (BECs) and cardiomyocytes (CMs). It plays an important role in lymphangiogenesis, angiogenesis and cardiac contractility by binding to its receptor APJ localized to LECs, BECs, smooth muscle cells (SMCs) and CMs. LEC-derived reelin aids the development of collecting lymphatic vessels and lymph propulsion by recruiting SMCs through MCP-1 upregulated in LECs that in turn respond to reelin in an autocrine fashion. SMCs recruitment also stimulates the release of reelin from LECs. Reelin then binds to its receptor Integrin-β1 to guide the CM function. LECs also secret adrenomedullin (AM) which not only binds to the receptor CRLR-RAMP2/3 and activates cAMP/PKA and PI3K/Akt pathways to protect CMs, but also mediates lymphangiogenesis via binding to CRLR-RAMP3 in LECs. MCP-1: monocyte chemotactic protein 1; CRLR: calcitonin receptor-like receptor; RAMP: receptor activity-modifying protein. The figure was created by using the illustration software on BioRender.com.

Alongside mediating developmental processes, crosstalk between cardiomyocytes and their surrounding vasculature is critical for protecting against injury-induced pathophysiological remodeling. Intercellular crosstalk between BECs and neighboring cells, or angiocrine signaling, is necessary for proper cardiac regeneration and function following ischemia/reperfusion (I/R) injury (Noireaud and Andriantsitohaina, 2014; Rafii et al., 2016; Colliva et al., 2020). BECs postnatally express tissue-specific signals to promote healing in the heart and various other organs without fibrosis (Rafii et al., 2016; Colliva et al., 2020). Platelet-derived growth factor AB, neuregulin-1, and endothelin-1 are among those angiocrine factors that protect cardiomyocytes from ischemic injury and restore cardiac function (Hedhli et al., 2011; Colliva et al., 2020). Apelin is a bioactive peptide expressed not only by endocardial ECs and BECs (Poirier et al., 2012), granulosa cells (Shokrollahi et al., 2022), T-cells (Yamada et al., 2023), trophoblasts (Mlyczynska et al., 2020), and fat cells (Yuzbashian et al., 2021), but also by newly formed lymphatic vessels after injury. It plays a critical role in angiogenesis, lymphangiogenesis, and cardiac contractility by binding to its receptor APJ localized to BECs, LECs, SMCs and cardiomyocytes (Wang et al., 2013; Tatin et al., 2017) (Figure 1). In a mouse model of myocardial infarction (MI), cardiac LECs involved in reparative lymphangiogenesis have been shown to express apelin and its receptor after ischemic injury, while nearby dormant lymphatic vessels do not (Tatin et al., 2017). Additionally, cardiac lymphatic vessels in apelin-deficient mice exhibit dilation and increased permeability post-MI (Tatin et al., 2017). The overexpression of apelin can stabilize the lymphatic vasculature under ischemic injury, indicating that apelin has a role in maintaining lymphatic vessel integrity and cardiac function after the onset of ischemia (Tatin et al., 2017). Clinical trials exploring regenerative bone marrow mononuclear cell (BMMC) transplants in patients with ischemic heart failure have revealed substantial improvements in cardiac function. Notably, a significant rise in plasma apelin levels accompanied these improvements post-transplantation, suggesting that the positive effects of BMMC transplantation are associated with its release of apelin (Gao et al., 2009). In fact, apelin can assist stem-cell-based therapies, as its overexpression can preserve the proliferative capacity of mesenchymal stem cells (MSCs) and restore their cardioprotective ability even after they have entered a senescent state (Zhang H. et al., 2021).

Numerous investigations and clinical trials have explored the treatment of cardiovascular conditions through angiogenic therapies, potentially paving the way for the application of lymphangiogenic therapies in the heart as well (Simons and Ware, 2003; Sabra et al., 2021). Following MI, phagocytes enter the infarcted area to remove dead cardiomyocytes that can then be replaced with fibrotic tissue (Klaourakis et al., 2021). Unfortunately, this new scar region is void of lymphatic vessels, creating a deficiency in immune cells and fluid clearance (Vieira et al., 2018; Houssari et al., 2020). Even minute changes in myocardial edema can further stimulate the development of fibrosis, increasing the rigidity of the myocardial wall that can lead to heart failure (Laine and Allen, 1991; Mehlhorn et al., 2001; Henri et al., 2016; Itkin et al., 2021). Studies in both ischemic and non-ischemic conditions, like dilated cardiomyopathy, have identified an endogenous response characterized by the upregulation of VEGF signaling, leading to an increase in lymphatic capillary density (Henri et al., 2016; Heron et al., 2023). Nonetheless, the accumulation of inflammatory factors in the affected area can cause adverse remodeling of the existing lymphatic network, manifesting in phenotypes such as a diminished number of collecting vessels and heightened vascular permeability (Henri et al., 2016; Brakenhielm and Alitalo, 2019). To assist the endogenous lymphangiogenic response, investigators have explored the augmentation of lymphangiogenesis through VEGF-C stimulation, which has been successful in reducing cardiac hypertrophy, restoring myocardial fluid balance, and overall improving heart function (Henri et al., 2016; Shimizu et al., 2018; Houssari et al., 2020; Song et al., 2020).

Regenerative medicine has been a major focus for ischemic heart disease treatment to allow for the complete recovery of cardiac tissue (Arjmand et al., 2021). Studies have been looking into cardiac tissue engineering paired with immunomodulatory materials that could offset the potential for tissue rejection, but the optimal way to prevent this would be to promote self-renewal (Arjmand et al., 2021). A recently published review outlines signaling molecules that are endogenously produced and coordinate cardiomyocyte proliferation and healing without fibrosis (Liu and Oliver, 2023). Creating adaptable lymphangiocrine factors that respond to the injured microenvironment or modulating their production within the microenvironment may allow for fibrosis-free cardiac tissue regeneration (Rafii et al., 2016; Liu and Oliver, 2023). Understanding the molecular and functional traits of these lymphangiocrine factors is important for discovering novel treatments for cardiovascular disease.

## Lymphangiocrine factors for cardiac development and maintenance

Although mammalian adult cardiomyocytes cannot undergo self-renewal, recent work has shed light on the interplay between cardiomyocytes and lymphangiocrine factors that are crucial for cardiac development and homeostasis (Figure 1). Cardiac lymphangiocrine factors are involved in processes that can prevent the accumulation of inflammatory factors, resolve edema, and impede the development of cardiac fibrosis. Liu et al. identified reelin as a significant factor for cardiac lymphangiocrine signaling (Liu et al., 2020). This discovery was made after observing an 80% decrease in reelin gene (*Reln*) expression in *Prox1* null embryos lacking lymphatic vessels (Liu et al., 2020). Reelin is a large secreted extracellular matrix glycoprotein that is expressed in astrocytes (Jandial et al., 2017), myeloma (Lin et al., 2017), hepatic stellate cells (Kobold et al., 2002), retina (Candal et al., 2005), neurons (Bottner et al., 2014), and other cells. It has been regarded as a key player in neural growth and migration and lymphatic vessel formation (Lutter et al., 2012; Alexander et al., 2023). By acting in an autocrine fashion, reelin expressed in LECs aids the development of collecting vessels and produces monocyte chemotactic protein 1 (MCP-1), a factor that recruits SMCs important for stimulating the secretion of reelin from LECs and for lymph propulsion (Lutter et al., 2012) (Figure 1). Although the source of SMCs remains unknown, MCP-1 is known for the recruitment of myeloid-monocytic cells (Fujimoto et al., 2009) which can harbor precursors of SMCs (Okano et al., 1990). While the study demonstrated the secretion of reelin from the conditioned media of cultured LECs by immunoprecipitation and western blotting (Liu et al., 2020), its actual concentration produced by LECs remains to be elucidated. Prior studies have designated very low-density lipoprotein receptor (VLDLR), apolipoprotein E receptor 2 (ApoER2), and integrin-β1(ITGβ1) as receptors for reelin, but only the ITGβ1 signaling pathway is significant for cardiac tissue growth (Liu et al., 2020; Alexander et al., 2023). After being proteolytic processed by SMCs that are adjacent to lymphatic vessels, reelin binds to ITGβ1 and potentiates downstream Akt and ERK activity that guides cardiomyocyte proliferation (Lutter et al., 2012; Adlung et al., 2017; Liu et al., 2020). The importance of reelin for cardiac development became evident when embryos with reelin-null cardiac lymphatics exhibited a decrease in heart size similar to that observed in pups ubiquitously lacking LECs (Liu et al., 2020). Both sets of embryos displayed a reduction in cardiomyocyte proliferation and a rise in cardiomyocyte apoptosis, suggesting a requirement for reelin-cardiomyocyte interactions to ensure proper cardiac development (Liu et al., 2020). In addition, the expression of reelin can regenerate cardiac tissue post damage, for wildtype MI-injured neonates showed improvements to cardiac function that were attributable to a resurrection of reelin activity within the infarcted area (Liu et al., 2020). While the delivery of exogenous reelin directly to the heart could not restore cardiomyocyte proliferation in adult mice post-MI, the resultant decrease in cardiomyocyte apoptosis and fibrosis confirms reelin’s cardioprotective role (Liu et al., 2020).

While reelin can restore heart function by preventing adverse cardiac remodeling after injury, its interaction with ApoER2 in the aorta can promote the vascular adhesion of leukocytes, contributing to the development of atherosclerosis (Ding et al., 2016; Calvier et al., 2021). Since the heart is reliant on its immune response to remove dead cardiomyocytes, initiate angiogenesis, and produce scar tissue, reelin-ApoER2 signaling may accelerate the accumulation of arterial plaque (Nahrendorf et al., 2007). Considering that atherosclerosis can further aggravate arrhythmia, a pathophysiological change associated with MI, it may be beneficial to therapeutically obstruct the interaction between reelin and ApoER2 (De Jesus et al., 2015; Ding et al., 2016; Calvier et al., 2021). Understanding the pleiotropic and potentially contradicting impacts of reelin on cardiovascular health can direct the formulation of therapeutic strategies and further clarify the mechanisms underlying heart disease.

A vasodilatory peptide and member of the calcitonin gene-related peptide family, adrenomedullin (AM or ADM) is another cardioprotective lymphangiocrine factor that has been shown to improve cardiac function (Janardhan et al., 2023) (Figure 1). The expression of AM in isolated lymphatic vessels from adult mice was found to resemble the expression of AM in the entire embryo at approximately E10-12.5 (Fritz-Six et al., 2008). During development, AM is expressed in the jugular lymph sac at E12.5 and is necessary for its proper growth (Fritz-Six et al., 2008). While AM is broadly expressed, including the cardiovascular system (Schonauer et al., 2017), it can serve as a useful biomarker for cardiovascular disease because of its elevated levels in the bloodstream of individuals with MI and heart failure (Nishikimi et al., 1995; Kobayashi et al., 1996; Hinson et al., 2000). Investigation into stem-cell-based therapy in a rat model of dilated cardiomyopathy (DCM) has found that both MSCs and BMMCs secrete significant levels of AM (Nagaya et al., 2005). Transplanted MSCs also enhanced cardiac function in the rat DCM model by provoking myogenesis and angiogenesis while preventing fibrosis, and these effects can be amplified by overexpression of AM in MSCs (Nagaya et al., 2005; Li et al., 2018). Alongside mitigating the high blood pressure associated with cardiovascular damage (Gibbons et al., 2007), AM can directly influence the composition of cardiac tissue by activating its receptors on cardiomyocytes to induce proliferation or on fibroblasts to reduce collagen production (Tomoda et al., 2001; Wetzel-Strong et al., 2014). Various mechanisms are responsible for mediating its diverse biological effects within the cardiovascular system. AM binds to the G-protein-coupled receptor (GPCR) calcitonin receptor-like receptor (CRLR), forming a heterodimer with a member of the receptor activity-modifying protein (RAMP) family (Yamauchi et al., 2014; Balint et al., 2023). Murine models with global genetic loss of AM, CRLR, and RAMP proteins have confirmed that this signaling mechanism is crucial for preventing jugular lymphatic vessel defects and edema (Fritz-Six et al., 2008). CRLR-RAMP signaling activates cAMP/PKA and PI3K/Akt pathways, which both have an antiapoptotic effect on cardiomyocytes (Nishida et al., 2008). AM-mediated cAMP/PKA signaling prompts earlier openings of mitochondrial Ca^2+^-activated K^+^ channels (mitoK_Ca_) (Nishida et al., 2008). This mechanism provides protection against apoptosis during ischemic conditions, and it has significantly reduced infarct area in previous I/R studies (Xu et al., 2002; Kato et al., 2003; Nishida et al., 2008). A rise in cAMP-mediated PKA activity also suppresses ERK activation, which is otherwise known to contribute to cardiac hypertrophy (Niu et al., 2004). This cardioprotective effect was confirmed when stress-induced mice lacking endogenous AM production showed greater left ventricular wall thickening as well as extensive fibrosis (Niu et al., 2004). Moreover, when AM was intravenously given to patients with acute MI, assessments showed a significant decrease in scar tissue and improvement in heart function just 3 months after treatment (Kataoka et al., 2010). Thus, under MI conditions, AM may prevent adverse cardiac remodeling by inhibiting fibroblast proliferation and collagen synthesis (Tsuruda et al., 1998; Horio et al., 1999; Tomoda et al., 2001). Myocardial I/R produces reactive oxygen species (ROS) that can kill cardiomyocytes and impair remedial LEC proliferation (Kato et al., 2003; Singla et al., 2022). ROS activates the PI3K/Akt pathway to confer cellular protection, but excessive ROS production can overwhelm the antioxidant capacity of the cell and cause cell death (Singla et al., 2022). Along with reducing ROS levels, AM administration at reperfusion was found to heighten P13K/Akt activation, thereby inhibiting destructive activities such as the mitochondrial release of proapoptotic molecules like caspase-3 (Kato et al., 2003; Weiss et al., 2003; Hausenloy and Yellon, 2004; Nishida et al., 2008). The additive effect of ROS and AM-mediated PI3K/Akt activation may safeguard against myocardial necrosis and excessive lymphatic permeability (Singla et al., 2022).

The distinct lymphangiogenic role for AM can be deduced from two questions (Potente and Makinen, 2017): which one of the three RAMP proteins is AM interacting with? (Petrova and Koh, 2018) at what developmental period is this interaction occurring? During embryogenesis, AM-RAMP2 interaction activates the MAPK/ERK pathway that regulates vascular growth (Fritz-Six et al., 2008). Without proper AM expression, embryos can die mid-gestation and present with cardiac and vascular defects such as abnormally small hearts and extreme hydrops fetalis (Caron and Smithies, 2001; Dackor et al., 2006). Meanwhile, AM-RAMP3 specifically mediates lymphangiogenesis in adults, suggesting that the AM-RAMP3 complex could be a promising candidate for addressing myocardial edema (Yamauchi et al., 2014). In fact, a mouse model that possessed AM gene (*Adm*) overexpression and received I/R injury showed a marked decrease in cardiac edema compared to controls, alongside an inexplicable sex difference in cardiac improvement (Trincot et al., 2019). Overexpression of *Adm* was also shown to increase mRNA levels of connexin 43 (*Cx43*) in cardiac LECs, directly improving lymphatic integrity (Trincot et al., 2019). Since Cx43 composes gap junctions that are involved in the transport of signaling molecules to nearby cells, AM treatment may also improve the channel of communication between LECs and cardiomyocytes (Zong et al., 2016). Further studies on the mechanisms of AM in disease states are necessary to mitigate cardiac and lymphatic remodeling seen in cardiovascular conditions.

## An overview of the intestinal niche

**FIGURE 2:**
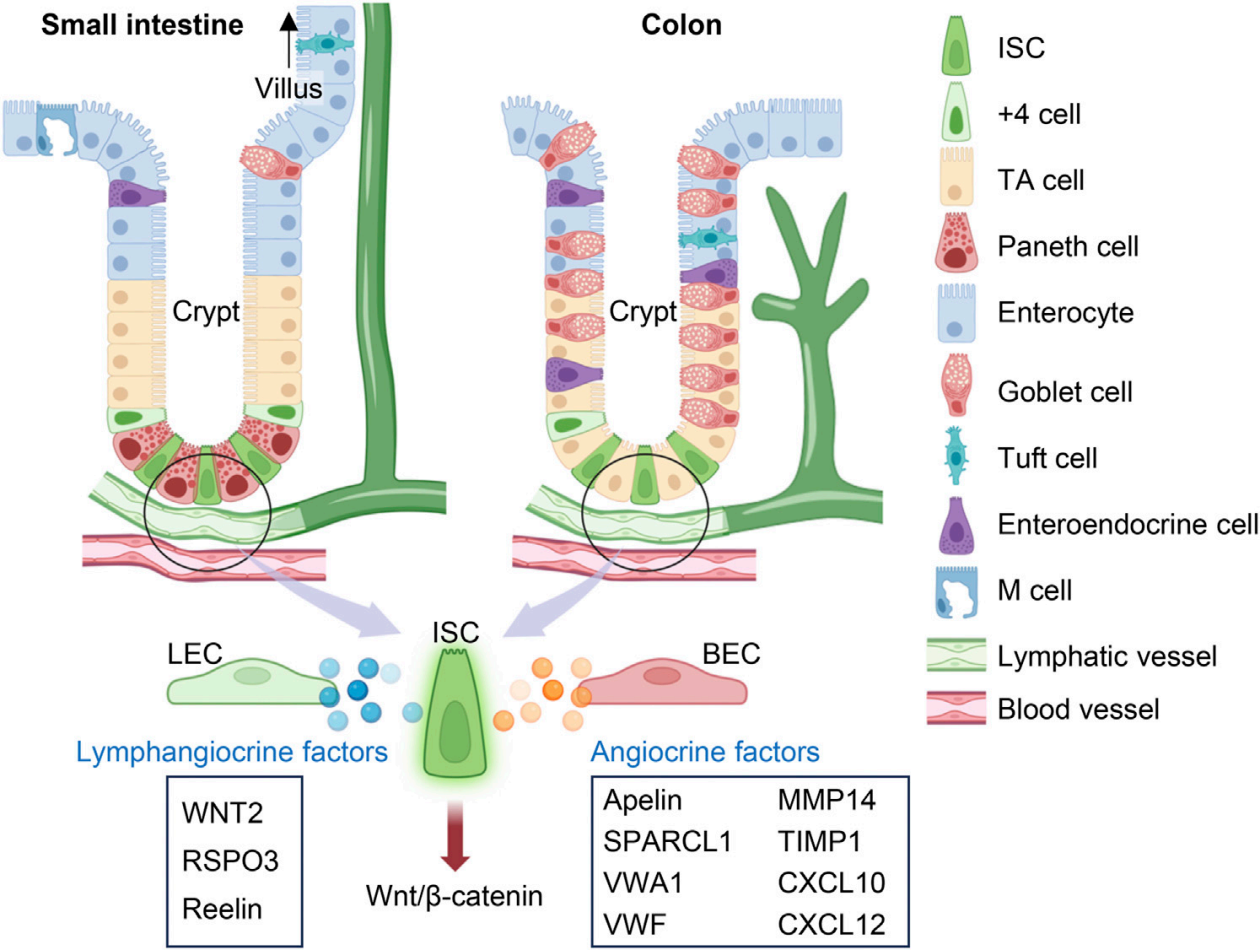
Lymphangiocrine and angiocrine factors in the intestine. In the small intestine and colon, intestinal stem cells (ISCs) are located at the base of crypts and can divide into transit-amplifying (TA) cells, which then mature into 6 kinds of terminally differentiated cells. Beneath the crypts lies the submucosal lymphatic and blood vessels. Lymphangiocrine and angiocrine factors derived from lymphatic endothelial cells (LECs) and blood endothelial cells (BECs) respectively play important roles in activating canonical Wnt/β-catenin signaling pathway in ISCs to maintain intestinal epithelium or assist epithelial regeneration after injury. The figure was created by using the illustration software on BioRender.com.

The gastrointestinal system contains multiple layers with distinct functions. The epithelium that encapsulates the small intestine and colon is a barrier that is constantly regenerating in response to its abrasive luminal environment and cues from the lamina propria (Gehart and Clevers, 2019). Present within the colon and at the base of each small intestine villus are crypts of Lieberkühn. Epithelial cells arise from intestinal stem cells (ISCs) located at the bottom of the crypt, migrating upwards along the transit-amplifying compartment as they mature into terminally differentiated cells including enterocytes, enteroendocrine cells, Paneth cells, goblet cells, tuft cells and M cells, each with unique absorptive and secretory behaviors (Potten, 1998; van der Flier and Clevers, 2009; Barker et al., 2012; Clevers, 2013; Gehart and Clevers, 2019; Ngo et al., 2022) (Figure 2). ISCs are responsible for the high rate of intestinal self-renewing activity. They are identified through the expression of the leucine-rich repeat-containing G protein-coupled receptor 5 (LGR5), a receptor for Wnt agonist R-spondins (RSPOs) (Moon et al., 2004; Goto et al., 2022). Canonical Wnt/β-catenin signaling replenishes the epithelial lining every 3–5 days, and its overactivation has been implicated in intestinal cancer (Krausova and Korinek, 2014; Kolev and Kaestner, 2023). Conversely, the inactivation of the Wnt signaling pathway substantially represses ISC proliferation and leads to the development of colitis (Kuhnert et al., 2004). Thus, the intestinal epithelium is strictly dependent on the Wnt pathway for maintaining homeostasis.

## Reparative processes in the intestines

### Inflammation modulation

Injury to the lining of the intestine triggers inflammatory reactions that precede tissue regeneration. Such injuries may result from infection, radiation, physical trauma, and pathologies that damage the intestinal epithelium, such as inflammatory bowel disease (IBD) and ischemic bowel disease (IB) (Richmond et al., 2018; Gonzalez et al., 2019). The ensuing inflammatory response by the lamina propria is necessary to eliminate bacterial invaders and initiate mucosal healing (Karin and Clevers, 2016). Immune cell-produced cytokines can directly enhance Wnt activity as well as other signaling pathways that can rescue the epithelium (Karin and Clevers, 2016). On the contrary, an uncontrolled inflammatory response can contribute to greater collagen deposition, upregulation of cell proliferative pathways, and increased apoptosis at the intestinal epithelial surface (Heller et al., 2008; Karin and Clevers, 2016). Since gastrointestinal pathologies such as IBD have been associated with faulty lymphatic structure and function (Van Kruiningen and Colombel, 2008; Bernier-Latmani and Petrova, 2017; Zhang L. et al., 2021), this can further impede the efficient clearance of inflammatory factors, facilitating the transition of IBD into colorectal cancer (Ocansey et al., 2020). Cases of chronic intestinal inflammation currently rely on immunosuppressive therapies to promote mucosal healing (Villablanca et al., 2022); however, this approach heightens the risk for infection and neoplasm (Singh et al., 2020).

### Angiogenesis

Angiogenesis is the process of developing new vessels from initial vessels and plays an important role in wound healing, chronic inflammation, and tumor occurrence, growth, and metastasis (Alkim et al., 2015). Inflammatory mediators have been found to initiate angiogenic processes that can contribute to the pathology of chronic inflammatory conditions (Alkim et al., 2015). Cases of IBD have demonstrated an imbalance between proangiogenic and antiangiogenic molecules, which can contribute to the development of abnormal blood vessel formation and further amplify inflammation (Carmeliet, 2003; Chidlow et al., 2007). Colorectal cancers can even exploit the communication between BECs and epithelial cells by amplifying the secretion of apelin from BECs, extending blood vessel density near the crypt to supply oxygen for pathological crypt expansion (Bernier-Latmani et al., 2022). On the other hand, animal models of intestinal disease have been used to emphasize the benefits of crosstalk action between BECs and the intestinal epithelial barrier in villi (Regensburger et al., 2021). Submucosal vessels transport nutrients, immune cells, and signals that bring forth adaptive changes to the epithelium (Miller et al., 2010; Clevers, 2013; Bernier-Latmani and Petrova, 2017; Tan et al., 2023a). Secreted protein, acidic, rich in cysteine-like 1 (SPARCL1), von Willebrand factor A domain containing 1 (VWA1), von Willebrand factor (VWF), matrix metalloproteinase (MMP)14, tissue inhibitor of metalloproteinases (TIMP)1, and C-X-C motif chemokine ligand (CXCL)10 have been identified as important BEC regulators of epithelial barrier integrity under inflammatory conditions (Sturzl et al., 2021) (Figure 2). BECs also express the angiocrine chemokine CXCL12 (also known as stromal-cell-derived factor, SDF-1), which can interact with either CXCR4 or CXCR7 receptors to regulate angiogenesis (Zhuo et al., 2012; Santagata et al., 2021). We recently found that intestinal BEC-derived CXCL12 can upregulate lymphangiogenesis as a mechanism for epithelial repair after I/R injury (Tan et al., 2023a) (Figure 2). More research should explore the crosstalk mechanisms between BECs and LECs in the intestine. Through further investigations on the intestinal microenvironment and how it assists epithelial regeneration, we can have a more complete picture of the mechanisms underlying intestinal disease.

### Mucosal healing

The intestinal niche consists of a vast array of cell types that can respond to intestinal injury and initiate mucosal healing. If the stem cell pool is compromised, newly differentiated cells can revert into ISCs, or the crypt may draw upon its quiescent reservoir of stem cells (Bmi1^+^, Msi1^+^, DCAMKL-1^+^) located at the +4 position of the crypt (Li and Clevers, 2010; Tan and Barker, 2014). Stemness is dependent on signals from the continuously evolving niche (Tian et al., 2011; Richmond et al., 2018; Harnack et al., 2019; Palikuqi et al., 2022a). The niche can coordinate tissue repair by secreting signals that bind directly with LGR5 to direct stem cell activity (Goto et al., 2022). Although Paneth cells surround ISCs and directly supply them with signaling factors, modifying Paneth cells so that they are unable to secrete Wnt has a negligible effect on ISC activity (Farin et al., 2012). New spatial transcriptomic techniques have been able to quantify the influence that surrounding mesenchymal cells have for maintaining the epithelial architecture, explaining their ability to compensate for the loss of epithelial-sourced ISC ligands (Duckworth, 2021; He et al., 2022; Niec et al., 2022). Mesenchymal constituents of the subepithelial niche that have been found to support ISC proliferation include immune cells, neural cells, pericytes, smooth muscle cells, myofibroblasts, telocytes, trophocytes, and endothelial cells (Meran et al., 2017; Gehart and Clevers, 2019; Duckworth, 2021; Palikuqi et al., 2022a). In particular, Foxl1^+^ GLI1^+^ platelet-derived growth factor (pdgfra)^high^ myofibroblasts (Singla et al., 2022), Foxl1^+^ CD34^+^ pdgfra^high^ telocytes (Stzepourginski et al., 2017; Shoshkes-Carmel et al., 2018), and CD81^+^ pdgfra^low^ Gremlin (Grem)1^+^ fibroblasts or trophocytes (Tsuruda et al., 1998) have been deemed as highly expressive of Wnt ligands vital for LGR5 signaling-mediation. Based on the developmental stage or extent of intestinal damage, the subepithelial niche can remodel to foster epithelial growth and restore organ function (Sphyris et al., 2021). In contrast, certain mesenchymal populations near blood vessels have been reported as having a profibrotic role (Di Carlo and Peduto, 2018). This raises an important question: which crosstalk mechanisms exist between subepithelial vessels and mesenchymal populations that assist epithelial regeneration?

## Lymphangiocrine factors that mediate intestinal epithelium regeneration

Epithelial self-renewal and differentiation rely on controlled signaling mechanisms to maintain intestinal homeostasis. Niec et al. recently found three lymphangiocrine signals that are highly expressed by lymphatic vessels adjacent to the crypt, one of them being WNT2 (Niec et al., 2022) (Figure 2). As a part of the 19 member Wnt family, WNT2 is a hydrophobic glycolipoprotein that complexes with LRP5/6 and Frizzled receptors, allowing for the translocation of β-catenin into the nucleus and ensuing stem cell proliferation (Clevers, 2006; van der Flier and Clevers, 2009; Carmon et al., 2012; Niehrs, 2012; Flanagan et al., 2018; Patel et al., 2019). A review on the gastrointestinal Wnt signaling pathway highlights its importance for intestinal crypt development and gastric epithelium regeneration (Flanagan et al., 2018). The benefit of WNT2 signaling is context-dependent. While deviant upregulation of WNT2 signaling has been implicated in the development of colorectal cancer (Xu et al., 2020), this mechanism has been beneficial for suppressing cellular apoptosis and mucosal inflammation (Liu et al., 2012; Valenta et al., 2016). Due to the short-range action of WNT proteins, LEC-derived WNT2 may be an indispensable source of ligands for ISCs in the crypt base (Farin et al., 2012; Farin et al., 2016; Niec et al., 2022).

Wnt requires interactions with RSPOs to amplify Wnt/β-catenin signaling and regenerate the intestinal epithelium (Niehrs, 2012). RSPO3, one of four RSPO proteins (R-spondin 1–4), has a high affinity for the LGR5 receptor found on ISCs (Carmon et al., 2011; Schuijers and Clevers, 2012). RSPO3-LGR5 associates with the Wnt-Frizzled complex and prevents its ubiquitination (Kim et al., 2008; Schuijers and Clevers, 2012). Instead, clathrin-mediated endocytosis internalizes this complex, facilitating the necessary induction of β-catenin for the transcription of target genes (Ohkawara et al., 2011; Niehrs, 2012; Ogasawara et al., 2018). While RSPO3 also has multiple sources within the intestinal niche, such as from Pdgfra^lo^Grem1^+^ trophocytes and Pdgfra^lo^CD81^−^stromal cells (McCarthy et al., 2020; Tan et al., 2023a), the loss of LEC-derived RSPO3 creates a significant reduction in intestinal regenerative ability (Clevers, 2006; Gubernatorova et al., 2016; Palikuqi et al., 2022b; Goto et al., 2022) (Figure 2). Control and *Rspo3* knockout mice maintain similar lymphatic density and lymphatic proximity with crypt epithelial cells; yet, after killing proliferative epithelial cells with chemoradiotherapy, Palikuqi et al. noticed that lymphatic specific *Rspo3* knockout mice had worsened intestinal recovery and less mucus production than controls (Palikuqi et al., 2022b). Graft *versus* host disease (GVHD) is a complication that can occur with bone marrow transplants and creates a severe inflammatory response injurious to epithelial cells in the gastrointestinal tract (Justiz Vaillant et al., 2023). GVHD lowers the number of LECs and their production of RSPO3, further aggravating damage to the gut epithelium and its mucosal barrier (Ogasawara et al., 2018). Treatment with an RSPO3 was found to protect the epithelium from GVHD damage and radiation-induced colitis by accelerating ISC proliferation (Bhanja et al., 2009; Takashima et al., 2011). Conversely, mutations that induce *Rspo3* genetic fusion are owed for the magnification of Wnt signaling causal of human colorectal tumorigenesis (Seshagiri et al., 2012; Conboy et al., 2021).

We have recently reported that FOXC1- and FOXC2-dependent transcriptional regulation of *Rspo3* in LECs is important for intestinal recovery from I/R injury (Tan et al., 2023a). Importantly, the gene-dosage effects of *Foxc1* and *Foxc2* in intestinal LECs are observed, based on the Chiu scores for the measurements of the degree of the impairments in intestinal regeneration (3 in LEC-double mutants vs. 2.5 in LEC-single mutants). Pre-conditioning with exogenous RSPO3 improved intestinal mucosal damage in LEC-specific double *Foxc1/Foxc2* mutant mice by upregulating regenerative activity at the epithelial barrier (Chiu scores, 3 in PBS-treated double mutants vs. 1.5 in RSPO3-treated double mutants) (Tan et al., 2023a). In an earlier study, treatment with RSPO3 prior to I/R injury was found to decrease immune cell infiltration into the lamina propria by maintaining tight endothelial adherens junctions (Kannan et al., 2013). I/R injury deprives an intestinal region of oxygen and later renourishes the tissue with oxygen to replicate the pathophysiology underlying a multitude of intestinal conditions that disrupt the gut barrier, including IB, necrotizing enterocolitis (NEC), IBD, and intestinal transplantation (Gubernatorova et al., 2016; Rumbo and Oltean, 2023). Hypoxic injury initiates at the villus tip and spreads down to the proliferative crypt (Shepherd, 1982; Blikslager et al., 1997), but the intrinsic regenerative ability of the intestine must keep up with epithelial cell death to prevent bloodstream susceptibility to bacteria (Williams et al., 2015). Initiated by a mucosal injury-event, the invasion of bacteria into the intestinal lumen can cause an inflammatory response that can rapidly progress into sepsis and multiorgan failure (Oldenburg et al., 2004). The optimal solution for intestinal ischemia without surgically removing intestinal sections or resorting to allogeneic transplants would be to modulate tissue regeneration using the patient’s own cells. Thus, evidence of epithelial barrier defects following detriments to Wnt/β-catenin signaling and improvements to intestinal damage after treatment with LGR5 ligands such as RSPO3 underscores the therapeutic role that lymphangiocrine modulation can have for epithelial proliferation.

The canonical reelin signaling pathway is not only expressed by myofibroblasts but also in crypt-associated LECs (Garcia-Miranda et al., 2013; Niec et al., 2022; Alexander et al., 2023) (Figure 2). Its receptors (VLDLR and ITGβ1) are expressed by cell types besides ISCs, positing that reelin has multiple effects on the intestinal niche (Niec et al., 2022). In addition, reelin may supplement ISC activities indirectly by activating cells that release stem cell trophic factors. Crosstalk between LECs and adjacent RSPO3^+^GREM1 fibroblasts (RGFs) jointly bolsters epithelial proliferation following irradiation damage to the mucosa (Goto et al., 2022). RGFs produce lymphangiogenic VEGF-D while LECs produce reelin, which binds to VLDLR receptors on RGFs and assists their release of RSPO3 (Goto et al., 2022). RSPO3 sourced from reelin-activated CD81^+^ crypt-bottom fibroblasts, or trophocytes, is a significant contributor to intestinal tissue repair (Chalkidi et al., 2022). Without RSPOs, Goto et al. showed that pre-treatment of RGFs with reelin still supports organoid growth, indicating their significant role in controlling ISC differentiation (Goto et al., 2022). However, different studies have reported conflicting accounts of the rate of epithelial proliferation in reelin (*Reln*) mutant mice (reeler) compared to control mice, expressing observations of both increased and decreased cellular migration along the crypt-villus axis (Garcia-Miranda et al., 2013; Niec et al., 2022). In terms of its presentation in intestinal disease, *Reln* is upregulated in genetic expression following DSS treatment, possibly serving as a protective mechanism against epithelial damage (Carvajal et al., 2017a). Chronic ulcerative colitis increases the likelihood of developing colorectal cancer, which explains why reeler mice not only had worsened colonic damage after DSS treatment but also a greater susceptibility to colitis-associated tumorigenesis (Carvajal et al., 2017a; Carvajal et al., 2017b; Alexander et al., 2023). Therapeutics that can modulate reelin production may better regulate the appropriate regenerative and quiescent ISC behaviors in the intestinal epithelium.

## Concluding remarks

Numerous studies have covered how cardiac and intestinal dysfunction impact different cell populations within the microenvironment. Crosstalk between lymphatic vessels and cardiomyocytes or epithelial ISCs is vital for returning these organs to their homeostatic state. Further understanding of crosstalk mechanisms between other cell types in the microenvironment is necessary to enhance our understanding of cardiac and intestinal pathology as well as spur the development of effective therapies. Current therapeutics focus on managing the associated inflammation and, in severe cases, are unable to restore the tissue without surgery (McLeod, 2003; Golia et al., 2014). Severe injury to either of these organs often necessitates organ replacement for full recovery. Additionally, damage to the lymphatic vasculature that follows cardiac and intestinal disease can impair the endogenous ability of the organ to clear inflammatory factors, contributing to tissue necrosis. Therapeutic strategies involving paracrine LEC factors show promise in regenerating the cardiac and intestinal tissue as well as restoring the lymphatic vasculature. Future work should address the various signaling pathways that LECs may use to communicate with members of the cardiac and intestinal tissue to uncover mechanisms that underly their pathology.
